# Patient Engagement as Measured by Inpatient Portal Use: Methodology for Log File Analysis

**DOI:** 10.2196/10957

**Published:** 2019-03-25

**Authors:** Timothy Huerta, Naleef Fareed, Jennifer L Hefner, Cynthia J Sieck, Christine Swoboda, Robert Taylor, Ann Scheck McAlearney

**Affiliations:** 1 Department of Family Medicine College of Medicine The Ohio State University Columbus, OH United States; 2 Department of Biomedical Informatics College of Medicine The Ohio State University Columbus, OH United States; 3 Division of Health Services Management and Policy College of Public Health The Ohio State University Columbus, OH United States; 4 CATALYST: Center for the Advancement of Team Science, Analytics, and Systems Thinking in Health Services and Implementation Science Research College of Medicine The Ohio State University Columbus, OH United States

**Keywords:** patient portals, health records, personal, health information technology, inpatient portals

## Abstract

**Background:**

Inpatient portals (IPPs) have the potential to increase patient engagement and satisfaction with their health care. An IPP provides a hospitalized patient with similar functions to those found in outpatient portals, including the ability to view vital signs, laboratory results, and medication information; schedule appointments; and communicate with their providers. However, IPPs may offer additional functions such as meal planning, real-time messaging with the inpatient care team, daily schedules, and access to educational materials relevant to their specific condition. In practice, IPPs have been developed as websites and tablet apps, with hospitals providing the required technology as a component of care during the patient’s stay.

**Objective:**

This study aimed to describe how inpatients are using IPPs at the first academic medical center to implement a system-wide IPP and document the challenges and choices associated with this analytic process.

**Methods:**

We analyzed the audit log files of IPP users hospitalized between January 2014 and January 2016. Data regarding the date/time and duration of interactions with each of the MyChart Bedside modules (eg, view lab results or medications and patient schedule) and activities (eg, messaging the provider and viewing educational videos) were captured as part of the system audit logs. The development of a construct to describe the length of time associated with a single coherent use of the tool—which we call a session—provides a foundational unit of analysis. We defined frequency as the number of sessions a patient has during a given provision day. We defined comprehensiveness in terms of the percentage of functions that an individual uses during a given provision day.

**Results:**

The analytic process presented data challenges such as length of stay and tablet-provisioning factors. This study presents data visualizations to illustrate a series of data-cleaning issues. In the presence of these robust approaches to data cleaning, we present the baseline usage patterns associated with our patient panel. In addition to frequency and comprehensiveness, we present considerations of median data to mitigate the effect of outliers.

**Conclusions:**

Although other studies have published usage data associated with IPPs, most have not explicated the challenges and choices associated with the analytic approach deployed within each study. Our intent in this study was to be somewhat exhaustive in this area, in part, because replicability requires common metrics. Our hope is that future researchers in this area will avail themselves of these perspectives to engage in critical assessment moving forward.

## Introduction

### Background

The use of ambulatory patient portals has substantially expanded since 2011, coincident with requirements for patient engagement placed on health care facilities as a component of meaningful use. Ambulatory patient portals allow patients to view their medical information, schedule appointments, manage their medications, and communicate with their doctors [1]. Research on ambulatory portals has found that patients want to adopt the use of these tools at a rate faster than that in which health care facilities have promulgated the technology [2]. Patient portals can foster increased patient engagement, and frequent users of outpatient health applications, portals, and personal health records show improvement in both risk factors for chronic diseases [3-7] and health outcomes [8,9]. As patient engagement technology shows positive results, some health care facilities have begun to explore other technologies that may improve the patient’s experience with the hope of impacting both clinical outcomes and satisfaction with care—inpatient portals (IPPs) are an example of such a technology.

Interest in IPPs stems from the benefits found in the use of outpatient portals as opposed to a response to regulation. Given the primary driver of health care costs at the national level is hospital-based care, facilities that can use technology to improve quality at a lower cost to the populations they serve—2 components of The Triple Aim [10]—are able to maintain a sustainable competitive advantage over their competition and, presumably, capture a greater component of the markets that they serve. An IPP provides a hospitalized patient with similar functions to those found in outpatient portals, including the ability to view vital signs, laboratory results, and medication information; schedule appointments; and communicate with their providers. However, IPPs may offer additional functions such as meal planning, real-time messaging with the inpatient care team, daily schedules, and access to educational materials relevant to their specific conditions [11,12]. In practice, IPPs have been developed as websites and tablet applications, with hospitals providing the required technology as a component of care during the patient’s stay.

Although studies that have explored the effect of IPPs in specific contexts go back to 2011 [13-15], adoption of large-scale commercial IPPs has generally been slow. One of the first such implementations occurred in 2013 at the Ohio State University (OSU) Wexner Medical Center with Epic Systems’ MyChart Bedside (MCB) product. As the first academic medical center to implement a system-wide IPP, OSU sought to use its unique position to document its journey and provide guidance on IPP adoption [1,11,12]. However, only recently has enough usage data been available upon which to explore outcomes through a *big data* approach—specifically through the analysis of audit log files.

Audit log files are server-side records of actions taken by a user. Every button pushed on a website or mobile app creates a record on the host computer, which can be used as the basis of an analysis. Log file analysis can be used to assess both when technology was accessed and what features of a program were used [16,17]. These log files are routinely assessed for operational purposes to help developers of websites and apps understand the behaviors of users to further improve these programs [18]. Log file analysis in the context of outpatient portals has been used to track the number of people using health applications in the patient portals; however, many of these studies measure only log-ins to the portal and not use of individual functions [6,8,19,20]. There has been limited research measuring the rate of use of one or two specific features of a tool, such as secure provider messaging or medication refill requests [21-23]. Log file analysis in the more recent IPP literature also shares similar constraints, where studies typically use crude measures to quantify IPP usage. These range from survey responses about use [24] to counts of overall use [25] or focus on the use of a few individual functions [26]. These kinds of studies report the total patients who used IPP and the total frequency of use across individual IPP functions; they typically do not report the limitations of using such measures.

The concept of use of a portal is multifaceted. For example, a researcher may define use in terms of the frequency with which a patient logs into the portal, or they may define use in terms of comprehensiveness, a function of the number of features a patient uses. Furthermore, although some may desire to measure use in a particular way, doing so may be hindered because of the manner in which the data are collected. As a result, the use of log files is not a straightforward task, and different definitions can be constructed to describe different elements of engagement. As a nascent area for research, there is limited scholarship and guidance on how one might approach the analysis of these data and explicate the challenges of analyzing log files.

### Objectives

This study sought to address 2 aims. Primarily, it provides a documentation of our approach to processing IPP log files using data from our IPP implementation in January 2014 through the subsequent 2 years ending and in January 2016. This time frame represents a period with relatively stable use and lower distortions caused by implementation issues. Although other studies have published usage data, these previous studies did not describe the challenges and choices associated with the analytic approach deployed within each study. Therefore, our primary goal was to address and explicate methodological issues one might find in a similar analysis of log files in an institutional local data setting. Furthermore, to foster a standardized approach to this research, we included our analytic files as appendices that can be used to analyze Epic patient portal log files. Our intent was also to provide guidance for future reviewers related to ensuring that studies adhere to the highest quality of data analysis.

The secondary aim of our study was to provide descriptive results from our institution’s data on IPP usage to present the implications of our methodological approach and the assumptions around the decisions we chose. Our results also provided a glimpse into the usage of the IPP and its functionality in the context of care and presented initial insights into how patients use this tool. The interpretation of our data could help to identify patterns that others may validate in their own patient populations, based on the implementation experience with our IPP. Altogether, this study sought to encourage research rigor and replicability with an eye toward informing practice based on the first large-scale implementation in place. We addressed the primary aim of our study in the Methods section, and the secondary aim has been presented in the Results section with the help of descriptive statistics.

## Methods

### Data Source and Data Model

IPP use information is extracted from a limited dataset composed of retrospective data on MCB use in the form of audit log files from the OSU Information Warehouse, secured under an Institutional Review Board approval. The data for this study were gathered between January 2014 and January 2016. Data regarding the date/time and duration of interactions with each of the MCB modules (eg, view lab results or medications and patient schedule) and activities (eg, messaging the provider and viewing educational videos) are captured as part of the system audit logs. All events are recorded as a triad of data that includes the following:

1. A medical record number (MRN).
2. An activity code (WPR 530: UA-TYPE), augmented by the extended information (WPR 550: UA–EXTENDED INFO).
3. A timestamp including the date and time (MDY HMS) when the activity took place (WPR 520: UA - INSTANT).

To safeguard the privacy of the patient, the MRN was replaced by a study identifier by an honest broker in accordance with institutional policy for research-related requests forming a limited data set. The activity code represents the action taken by the patient (eg, a log-in, a log-out, accessing education materials, or sending a secure message). The parentheticals provide the variable name in the Epic data model for ease of identification. Other data sets that we merged with our audit log files are admission, discharge, and transfer (ADT) data; patient summary; and hospital charge data.

**Figure 1:**
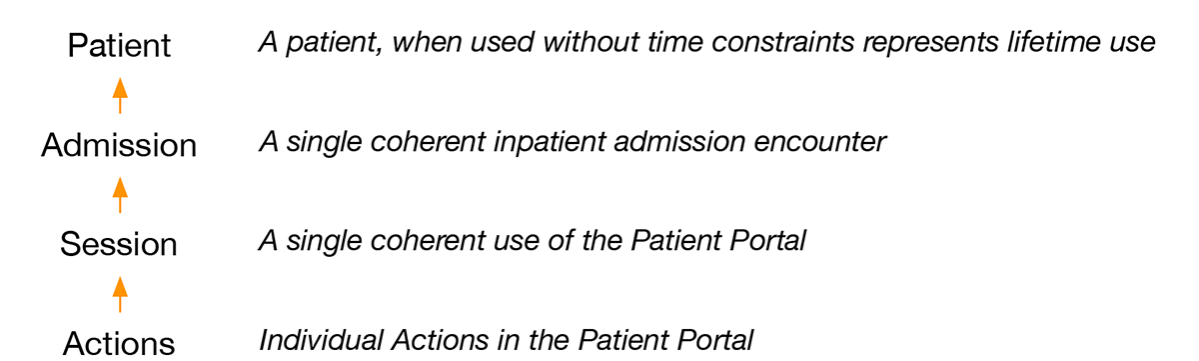
Data aggregation model.

Our data model was primarily guided by 2 key objectives that determine the dimensionality of our final data set: (1) level of analysis of IPP use and (2) measure of IPP use. The level of analysis of IPP use indicates the rows of the data set, and the measure of IPP use represents the columns of our dataset (see Figure 1). From a conceptual standpoint, the level of analysis can be viewed as (1) session: a continuous period of portal use from the moment a patient logs into the IPP to the moment they logout; (2) admission: a period of continuous use during a clinical encounter, from admission to discharge from a unit; and (3) patient: all IPP use for a given patient across all inpatient encounters within the medical center. Similarly, the measure of IPP use can be viewed as (1) frequency: the count of use or the count of a particular action over a level of analysis; (2) comprehensiveness: a count of the number of unique actions or the number of unique activities over a level of analysis; and (3) duration: the amount of time spent using the portal over a level of analysis. Other important concepts that need to be considered for our data model are presented in Table 1.

**Table 1:** Other important concepts related to the data model.

Concept	Description
User action or task	The recorded server actions that appear in the audit logs. Some represent an intentional action of the patient, whereas others represent background functioning of the portal (eg, an action will be recorded in the log file if the information on a given page is refreshed as the patient is viewing it).
Function	A group of user actions or tasks.
Provisioning	When the patient is provisioned with the hospital tablet, within a hospital encounter. This marks the point at which the portal is first available to the patient.
Length of stay	The number of calendar days a patient was in the care of the medical center.
Encounter	A patient’s interaction with the medical center.
Contact serial number	A unique identification for each patient encounter.

### Data Processing

The approach we deployed to process our data to structure it to fit our data model objectives described in the previous section involved 6 overarching modules. For each of these modules, we explicated their goals and the critical assumptions around the data processing below.

### Module 1

Process raw ADT dataset (refer to Stata Module 1 in Multimedia Appendix 1): The goal of this module was to establish the start and end date of each specific inpatient clinical encounter during which a patient received continuous medical care. To achieve this, we first inspected the ADT patterns for a patient at a facility within OSU using the ADT data set. For the data to fit our definition of a clinical encounter or admission, we performed 2 transformations of the raw ADT data:

- Combine overlapping encounters: Patients may have more than one recorded ADT event in the raw data within a time period. For example, a patient may be admitted for an inpatient stay, admitted to another hospital within the system for a procedure, and then return to the original room. The result is multiple admissions but a single discharge. We treated these as a single admission.
- Combine adjacent encounters: Patients may have encounters that occur within a short time frame of each other. This is generally attributable to patients moving between different OSU facilities. Without a way to differentiate true discharges from transfers between facilities, we merged all encounters that occurred within 4 hours of each other (which covers approximately 80% of all transfers at our institution) to form a single admission event.

### Module 2

Process raw hospital charges dataset (refer to Stata Module 2 in Multimedia Appendix 2): The goal of this module was to obtain information about patients’ clinical diagnoses. As MCB is an IPP, initial processing of the hospital charges’ data requires that data be restricted to only inpatient encounters (ie, excluding any instances of outpatient or emergency visits). As a single hospital charge may cover multiple encounters, we created a new variable: *hospital account id*, which links specific charges to an admission period. An artifact of creating this variable is the presence of duplicate *hospital account ids* with different admission and discharge times in the raw source data. As we used the raw ADT data to define admission times, we retained only one of the duplicate observations of *hospital account id* in the hospital charges files to obtain information about patients’ clinical diagnoses and validated that this information was indeed the same across the duplicate observations.

### Module 3

Process raw Audit Log dataset (refer to Stata Module 3 in Multimedia Appendix 3): The goal of this module was to apply existing category labels for the MCB function to users’ actions. A review of raw audit log data found that the log files include activity codes, which are generated by the computational environment that represents administrative actions not initiated by the patient (eg, pushed data to ensure that the tablet does not display stale data). A review of system documentation, conversations with the implementation team, discussion with the vendor (Epic), and subsequent data visualizations were used to assist in the identification of data in the activity code associated with the idiosyncratic choices of the institution in its implementation, and those actions were removed from the analysis. We removed 2 user actions: (1) Get Menu Items and (2) Get Provider, as the former was a navigational action and the latter appeared as multiple separate actions, one for each Care Team member a patient has, whenever the Get Care Team action was performed. We also removed Get Wallpaper Data actions, which represented a page refresh action and occurred every 5 min (discussed in greater detail in Module 5).

Our approach resulted in 9 MCB functions (see Table 2). Analysis of the raw data also found that the logout and login variables are not reliable indicators of a contiguous MCB use period. Hence, we did not use these variables for our classification of users’ actions. Table 3 provides the description and use case examples for each of the 9 functions identified by our study team.

**Table 2:** Active and inactive MyChart Bedside users’ actions.

Functions	Users’ action	Activity
Access educational materials	Get 1 patient-prescribed education title	Active
	Update education status	Active
	Get patient-prescribed education titles	Not active
Access personal notes	Create patient note	Active
	Delete patient media	Active
	Delete patient note	Active
	Update patient note	Active
	Get patient notes	Not active
Administrative	Identify user with lock	Active
	Make Bedside link	Active
	Send MyChart email	Active
	Accept terms and conditions	Not active
	Handshake	Not active
	Load terms and conditions	Not active
	Set lock for user	Not active
	Update photo for user	Not active
	Login/logout	Not active
Check secure messages	Get messages	Active
Happening soon	Create user-created event	Active
	Delete user-created event	Active
	Get appointment event detail	Active
	Get medication administration details	Active
	Get surgery event detail	Active
	Load schedule	Active
I would like	Delete patient request	Active
	Save patient request	Active
	Get patient requests	Not active
MyChart Ambulatory	Create MyChart account	Active
	Load MyChart info	Active
	Validate MyChart login	Not active
	Validate MyChart password	Not active
Review current care team	Get Care Team	Active
Review vitals and lab results	Get lab result comments	Active
	Get health metrics	Not active
	Switch bedside admission	Not active
Send a secure message	Save message	Active

**Table 3:** MyChart Bedside functions.

Function	Description	Use case example
Happening soon	Review scheduled upcoming tests or procedures	The patient wants to see what time they can expect the respiratory therapist to come for their breathing treatment.
I would like	Request one of a number of ancillary services	The patient wants to see a representative from Pastoral Care.
MyChart Ambulatory tasks	Access the ambulatory patient portal through the MCB^a^ conduit	The patient wants to create an outpatient MyChart account or change the password on an existing MyChart account.
Review vitals and lab results	Review vital signs including blood pressure, heart rate, and temperature	The patient wants to see the results of their morning blood tests.
Send a secure message	Send a secure message to the care team	The patient wants to ask a question about their treatment.
Check secure messages	Check whether a secure message has been received	The patient wants to see whether a member of the care team has responded to their earlier question.
Access personal notes	Record and review personal notes (audio and written)	The patient has a question for their doctor that they want to remember to ask during rounds.
Review current care team	Review active members of the care team	The patient wants to find out the name of the nurse that just came in, but was too shy to ask.
Access educational materials	Access training materials through a link to an external health information content provider	The patient was assigned educational material by the care team. The care team will engage in teach-back once the patient is done or will ensure the content is discussed no later than 24 hours after assignment.

^a^MCB: MyChart Bedside.

**MyChart Bedside external actions.**

External actions:

- Dining on demand
- Welcome video
- Getting started
- MyChart Bedside (MCB) patients’ rights and responsibilities
- MCB patients’ tutorial

In addition to the features available directly through MCB, the hospital also provides external tools, such as the ability to order food, through the portal. These external actions are identified in the log as *Media/Web content*, which the software uses for any remote internet call. The *extended info* variable (WPR 550) provides a unique code that identifies each of these external actions. Observations with these actions were replaced with specific MCB active tasks (see Textbox 1).

In some cases, multiple login events appeared sequentially, with no other actions recorded. It was determined that these did not provide any meaningful information, and all but one of these sequential logins was dropped. There were also instances where the same actions occurred multiple times with the same time stamp. We retained only one observation in such a case, although it was difficult to delineate 2 close observations in time as they may truly be unique or artifacts of system idiosyncrasies.

### Module 4

Merge ADT, Audit Log, and Hospital Charges data sets (refer to Stata Module 4 in Multimedia Appendix 4): The goal of this module was to first link the data from the processed ADT and audit log data sets and then to link the new dataset with the Hospital Charges data set. As there was no unique identifier across the ADT and audit log data sets, we linked our data sets by matching all the records using the patient’s MRN and keeping only the audit log observations that fell within a clinical encounter period. This process did yield some log observations that fell outside of a clinical encounter, and we dropped these observations in our linked data set. The dropped observations fell into 2 general categories, those that were (1) very close to encounter periods and (2) far from encounter periods. The former may be the result of potential inconsistencies in our session variables, and the latter phenomenon is harder to diagnose as use may appear days or even years after being removed from any patient encounter. These observations were removed from the data. Finally, we used the *hospital account id* from the ADT and Hospital Charges data sets to link the hospital charges file to our merged data set.

### Module 5

Generate levels of analysis (refer to Stata Module 5 in Multimedia Appendix 5): The goal of this module was to create variables to flag different temporal dimensions within which IPP use could be measured. Our first step was to create a variable that flags the beginning of each session. We defined a session to be a continuous period of portal use without 15 min of inactivity. It should be noted that our institution logs individuals out of the system after 10 min of inactivity; however, a review of the data showed the logout system was not always effective. As a result, we chose 15 min as a conservative estimate to represent sufficient inactive time to justify the end of a session. Using our definition of a patient admission, established in Module 1, we also created a variable to flag unique patient admissions. We were also able to identify unique patients who used the IPP by their MRN.

**Figure 2:**
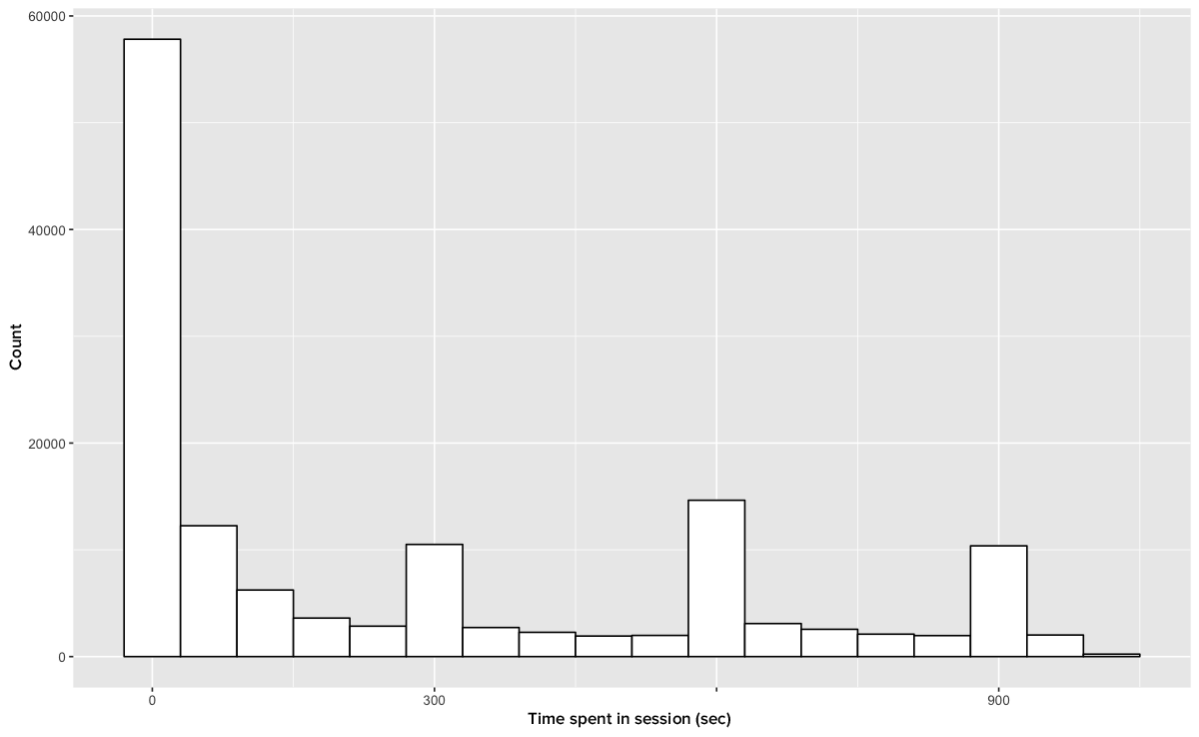
Session length (n=139,181).

**Figure 3:**
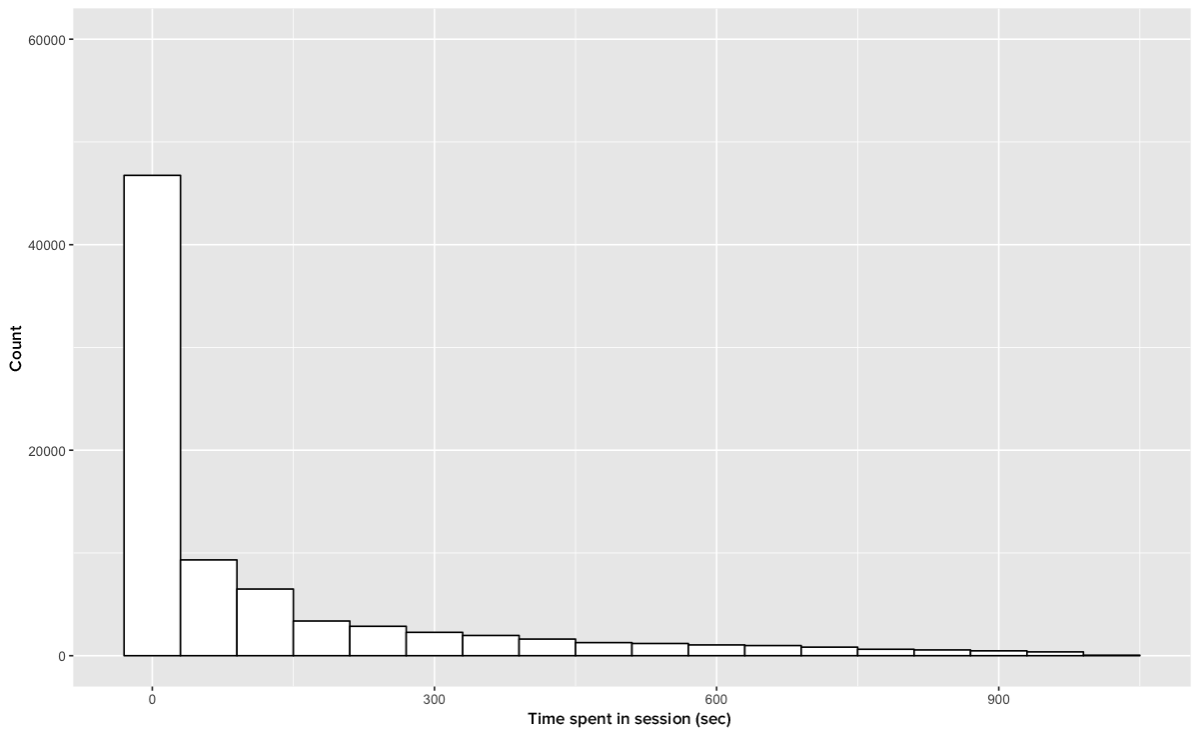
Session length (n=82,117).

With respect to IPP sessions, we encountered additional idiosyncrasies in the data that required processing. As previously noted, the Get Wallpaper Data action resulted in page refreshes at intervals of 5 min. Figure 2 illustrates this phenomenon with session data, and Figure 3 illustrates the same data after we omit this action.

### Module 6

**Figure 4:**
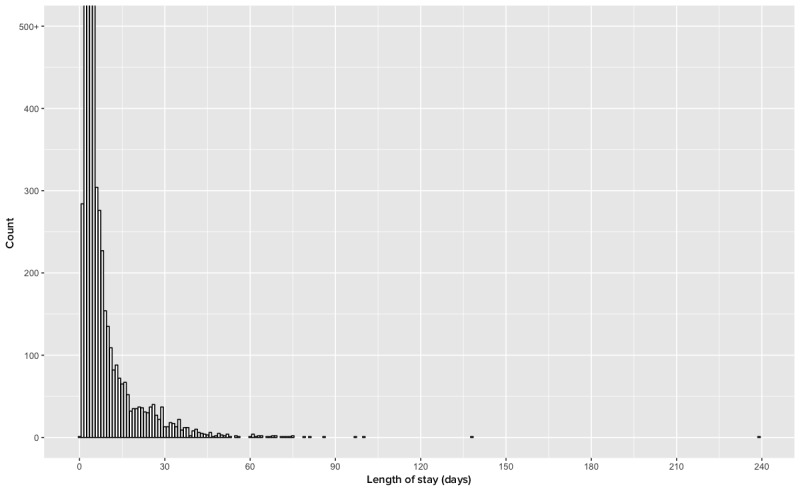
Length of stay (n=6575).

Generate measures of use (refer to Stata Module 6 in Multimedia Appendix 5): The goal of this module was to create variables to measure IPP use from several different dimensions. As we attempted to develop our use measures, we first encountered challenges with patients using MCB who had extremely short or long lengths of stay (LOSs). Figure 4 resents the LOS associated with MCB users. The data approximate a log normal distribution; however, a small number of provisioned patients were long-term admissions to the hospital. Given the disproportionate effect of outliers on descriptive statistics, we sought to identify a point in the distribution where we no longer felt that we had sufficient data to make robust approximations. In the OSU case, we did not include individuals who were hospitalized longer than 30 days as the number of patients at that point fell below our *a priori* group size of 30 (indicating that at least 30 people had that total LOS). As we collected more data over time, we expected to push this boundary into longer LOSs.

Additionally, as illustrated in Figure 4, we identified another artifact in the audit log data. In cases where an individual logs in to review their current status on the home screen, the result is a *zero-time session*, one in which no additional functions are activated. If the goal is to create a metric for the duration of time the patient uses the tools, then that measure of duration would be systematically biased. As a result, the development of a metric of *duration*, defined as *the time spent in a particular session*, was determined to be unable to be constructed using audit log files from MCB. Hence, we focused on the creation of IPP use measures of frequency and comprehensiveness measures.

Furthermore, provisioning of the tablet with MCB occurs subsequent to admission and, as a result, the LOS and the number of provision days may not be equivalent. This was an important consideration because the use of LOS as a comparative construct creates systematic bias in the result. Consider a hypothetical patient who presented in the emergency room, was deemed nonresponsive, and was then admitted to the hospital. It may be that the patient becomes coherent on day 3 and provisioned with the IPP on day 5, based on established protocols. This difference between day first provisioned and LOS was visualized to assess the practice pattern associated with the provisioning process.

**Figure 5:**
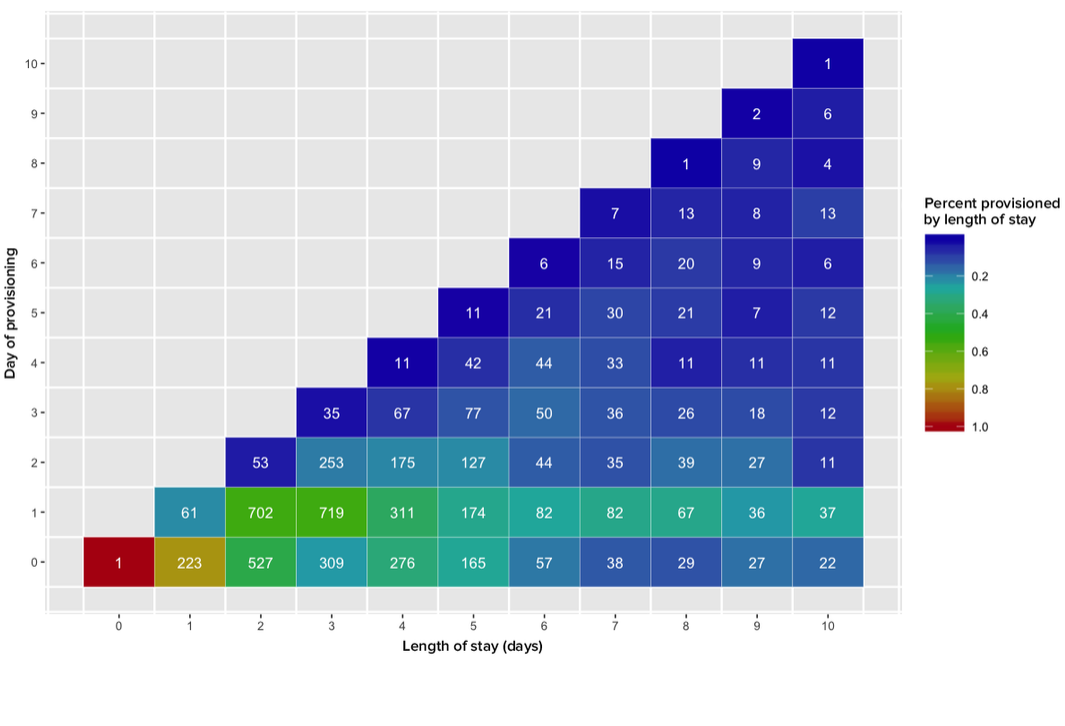
Day of provisioning by length of stay (n=5418).

Each tile in Figure 5 represents the intersection of a patient’s LOS on the *x*-axis with the day they were provisioned a tablet during that admission on the *y*-axis. In the context of this graph, LOS is constructed such that if a patient is admitted and discharged on the same day, their LOS would be equal to zero and is not based on an hourly calculation of days. The number in each tile represents the total number of patients at a given intersection of LOS and day of provisioning. For example, 719 patients had an LOS of 3 and were provisioned a tablet on the second day (day of provisioning=1 in Figure 5). The color gradient represents the percentage of patients provisioned on a given day within each LOS. It should be noted that if individuals were always provisioned on the day they were admitted, the graph would be the line of red tiles at the day of provisioning zero. What we see in fact is that there is significant variability in the time from admission to tablet provision. It may take several days for a patient to get their tablet and, as such, LOS as the basis of analysis may suffer from distortions associated with provisioning practice.

The research team also identified a number of exogenous factors that impacted the use of the IPP. For example, tablets are assigned to the patient while in a unit and, as a result, the patient could experience a discontinuity in access attributable to a change in unit, as was commonly the case when a patient was moved between units after a procedure (eg, prepartum to postpartum). Figure 5 focuses on the first 10 days because over 90% of all patients are provisioned for 10 days or less. As a result, the presentation of data in tables using the first 10 provision days represents a relatively robust approximation of usage for the majority of patients.

As a result of our processing, although duration, frequency, and comprehensiveness could offer a holistic view of IPP usage, only the latter 2 of those measures are robust. We have presented the preliminary results of this application of our methods in the next section using the frequency and comprehensiveness measures of IPP use.

## Results

### Overview

We applied our methodology for processing and merging the data sets described in the Methods section to MCB use data at OSU for the time periods between January 2014 and January 2016. Table 4 provides context for our study sample in relation to the patients seen by the OSU general patient population. Our sample of MCB users consisted of patients who were generally younger, more likely to be female, and had similar Charlson Comorbidity Index scores (range 0 to 19) to the OSU general population. They also had a slightly lower count of diagnoses than the general population.

The results below enumerate the implications of our decisions in the previous section, provide a glimpse into the usage of the IPP and its functionality in the context of care, and present initial insights into how patients use these tools. We offered the interpretation of our data so as to identify patterns that others may validate in their own patient populations, based on the implementation experience with our IPP. The results we presented below are based on the first 10 tablet provision days within an admission.

### Frequency

Table 5 presents descriptive statistics on the frequency of IPP use across the various MCB functions based on our 3 levels of analysis: (1) session, (2) admission, and (3) patient. With respect to session, our results indicated that the median number of active tasks across sessions for all MCB functions was zero. This suggests that the majority of sessions contained only administrative/navigational tasks. We found that the top quartile for Happening Soon and View Care Team had 2 or more active tasks. Within a session, we found that the Send a Secure Message function was rarely used, albeit the Check Secure Messages function was used relatively more. The View Care Team function was the most popular function used. As previously noted in the Data Processing section, variables such as View Care Team may be inflated because of how the observations were recorded. For the admission and patient levels of analysis, the View Care Team and Happening Soon functions appear to be used more than the other MCB functions. These 2 functions also had a high level of spread based on their interquartile ranges. Like the session level, the Send a Secure Message was the least used function. Across the statistics, it is important to recognize that the admission and patient levels of analysis contain a high level of variability because of, for example, different numbers of admissions for a patient.

**Table 4:** Summary statistics of patient characteristics.

Characteristics	Study sample (n=5305)	Ohio State University general population (N=69,761)
Age (years), mean (SD)	45.06 (16.91)	53.64 (18.01)
Female (%)	75	55
Charlson Comorbidity Index, mean (SD)	3.06 (3.55)	2.77 (3.12)
Diagnoses, mean (SD)	13.05 (8.98)	15.49 (9.40)

**Table 5:** Frequency of MyChart Bedside functions.

Functions	Sessions (n=59,802*)*		Admissions (n=6105*)*		Patients (n=4979*)*	
	Median	IQR^a^ (Min-Max)	Median	IQR (Min-Max)	Median	IQR (Min-Max)
Happening soon	0	2 (0-170)	6	19 (0-1011)	6	21 (0-3562)
I would like	0	0 (0-11)	0	1 (0-29)	0	1 (0-50)
MyChart Ambulatory tasks	0	0 (0-23)	0	1 (0-42)	0	1 (0-48)
View vitals and lab results	0	0 (0-60)	1	4 (0-226)	2	4 (0-312)
Check secure messages	0	0 (0-187)	1	3 (0-190)	1	3 (0-190)
Send a secure message	0	0 (0-4)	0	0 (0-12)	0	0 (0-12)
Access personal notes	0	0 (0-11)	0	1 (0-34)	0	1 (0-42)
View care team	0	2 (0-191)	6	13 (0-536)	6	14 (0-1963)
Access educational materials	0	0 (0-44)	1	2 (0-67)	1	3 (0-86)

^a^IQR: interquartile range.

**Table 6:** The number of distinct MyChart Bedside functions used.

Number of functions	Sessions (n=59,802), frequency (%)	Admissions (n=6105), frequency (%)	Patients (n=4979), frequency (%)
0	26,257 (43.91)	494 (8.09)	324 (6.51)
1	8,259 (13.81)	278 (4.55)	211 (4.24)
2	11,450 (19.15)	568 (9.30)	425 (8.54)
3	6041 (10.10)	651 (10.66)	519 (10.42)
4	3697 (6.18)	905 (14.82)	704 (14.14)
5	1766 (2.95)	813 (13.32)	633 (12.71)
6	945 (1.58)	588 (9.63)	485 (9.74)
7	852 (1.42)	663 (10.86)	576 (11.57)
8	533 (0.89)	1033 (16.92)	975 (19.58)
9	2 (0.00)	112 (1.83)	127 (2.55)

### Comprehensiveness

Table 6 presents the descriptive statistics on the count of unique IPP actions performed by patients based on our 3 levels of analysis. As the session level, the results concur with our analysis using the frequency measure in the sense that the most common activities were administrative/navigational tasks. The admission and patient levels of analysis both reflect a similar pattern, where there is an even distribution between 2 and 8 functions. At 8 functions, we also found that the Check Secure Messages function was the least commonly used task. It should be noted that, across all levels, the proportion of data for using all 9 functions was very low.

We generated similar statistics for the frequency and comprehensive measures using the first 30 days within an admission (see supplementary tables in Multimedia Appendix 6). The patterns in these results did not vary significantly from the use measures presented using the first 10 tablet provision days.

## Discussion

### Summary

IPPs offer patients access to unique features not required in outpatient portals. Although significant research has been done to study outpatient portals, the nascent nature of IPPs lends them to exploration. As we noted in the Introduction section, the purpose of this study was twofold. The goal with our first aim was to address and explicate methodological issues that one might find in a similar analysis of audit log files from an institutional local data setting. Our intent was to provide guidance for future reviewers related to ensuring that studies adhere to the highest quality of data analysis. The second aim of our study was to offer a sense of the implications of our methodological approach, provide a glimpse into the usage of the IPP and its functions in the context of care, and present initial insights into how patients use these tools. Our interpretation of the data could help identify patterns that others may validate in their own patient populations.

With respect to the first aim, we provided 6 modules that described the goals and critical assumptions around our methodological approach. We supplemented these discussions with the Stata code (available as appendices in the Multimedia Appendix 1) used within each module so that researchers can apply them to their own data. In addition, below we have highlighted 4 key lessons that we learned related to this aim. These lessons should guide and inform future research using audit log files with the intention of helping researchers overcome many of the complex, tedious, and resource-intensive tasks involved with parsing through and combining this type of data [27]. Our hope is also that future researchers in this area will avail themselves of our perspectives to engage in critical assessment moving forward.

### Lessons Learned

First, as the precision of recording of IPP user activity in the audit log files is imperfect, significant experience and familiarity are needed with the data to trace the flow of user activity [27-29]. To address this major challenge, we recognized, very early in the process, the need for a data model with prime directives that would enable us to reframe or restructure the data for the purposes of our study. This was especially important for our study given that the original intent of the data collection was not for research purposes but rather for our institution’s operational needs. We found that explicating the levels of analysis and the measures of use were 2 useful objectives around which our data needed to be redefined. The need for strong assumptions around the data model (eg, constraining our analysis to 10 or fewer IPP provision days) was also necessary.

Second, we found that using different levels of analysis involved information that contained both unique and common aspects. Assessing IPP use from the session to the patient level provided a view of IPP utilization that ranged from a granular to a more aggregate perspective. Notably, our data indicated that IPP use at the admission level was strongly correlated with the relatively longer-term view of use at the patient level. This phenomenon needs to be further explored to confirm that this pattern exists beyond our institutions’ data.

Third, and as other researchers have noted [30-33], we found that the choice of a specific IPP measure of use is complex and involves tradeoffs. The use of frequency provided us a count of the unique actions within a level of analysis, albeit the number was sensitive to patients’ LOSs (as noted by the extreme maximum points in our descriptive statistics for this measure). Although the comprehensiveness measure reflected whether patients used all the active IPP functions, this measure does not demonstrate users’ intensity of use of specific functions.

Finally, we recognized the utility of higher-level categorizations of the over 30 IPP user actions. By making these data parsimonious, we were able to effectively analyze and generate statistics about the data that may have been more tedious and complicated by the many types of user actions. We, nonetheless, recommend that future research explore other approaches to studying users’ actions to be able to generate robust findings about IPP use. These approaches may include techniques such as clustering, Gaussian mixture models, and multidimensional scaling.

With respect to the second aim, we offered an interpretation of our data based on the implementation experience with our IPP to identify patterns that others may validate in their own implementations and within their own patient populations. The analysis primarily demonstrates how the decisions we made around our data model’s assumptions and the methodological steps we undertook influenced the presentation of the output for our results; this subsequently could determine the narrative used by researchers and practitioners to communicate the patterns of IPP user actions found in their data.

As organizations move forward with their implementation of these tools, the need for benchmarks is critical to understand the relative success or challenges of a specific implementation. We also noted that use of the technology is a long-tailed phenomenon impacted by the presence of different types of users. For instance, 1 user, who was provisioned for less than three days, viewed members of the care team 111 times. In practice, wide differences in use may stem from a number of reasons, including comfort with technology as well as a patient’s disease state. Future research should attempt to identify the factors that contribute to distinguishing superusers (highly proficient users), hyperusers (high-frequency users), and intermittent users. In this instance, on provision day 1, 1 hyperuser had 41 separate sessions. To this end, we presented data in our results with both the interquartile ranges and the minimum and maximum to allow researchers to see the need for a classification schema moving forward.

### Limitations

Key limitations should be noted with respect to our experience working with IPP audit log file data. Although we presented our methodology as a general approach to parsing and analyzing log file data, there are idiosyncrasies to such data that may exist specifically to an institution or the source of the data. It is impossible to consider all of these contingencies, but researchers should be mindful of the ones we have listed—along with the possibility of others—when using our approach.

Another limitation involves potential confounders that can influence the results presented. For example, the use of whiteboards as a means to communicate care team member information, changes to the care team members during a patient encounter, the times/days a patient uses the IPP, and the facility/unit within which the IPP is used may all influence the patterns identified in our data. However, this type of analysis is beyond the scope of our preliminary analysis and lends itself to future explorations that need to capture all of these and other possible contingencies in IPP use.

Finally, we submit that there may be other decisions that may be relevant or undiscovered with respect to how log file data need to be parsed or analyzed; these may have been potentially overlooked or they may be functions of the time and experience we have had with our data. Our hope is that we have motivated a conversation to advance future research to uncover these important aspects of the data that can help further improve the methodology used to manage these data.

### Conclusions

This study represented the first volley into the IPP space by studying the experience of a health care system that has fully integrated the technology into its care processes. As we explore these data moving forward, we expect to offer some of the first glimpses into how such systems will be used in the context of care. Models for behavior change, such as the Health Belief Model [34], propose that systems such as IPPs may support patient engagement through education provided via the portal, increasing patients’ knowledge about their condition as well as helping them assess the benefits of and barriers to taking action. However, our results would suggest that the longer the patient is in the hospital, the less likely they are to use the IPP to gain a greater understanding of their condition. Furthermore, although patients and providers can use the IPP to exchange information and educational messages can be tailored to patients’ needs, it would seem that these phenomena are not yet happening in practice.

Patient portals offered in the inpatient setting present patients with targeted education and tools for managing health at a time when their perception of the threat from not managing their health is likely to be high, and therefore they would be more likely to engage with the technology [35]. Information and technology alone, however, are insufficient to fully engage patients in their care—patients also need motivation to engage [36-40]. A common element of health behavior change theories is the need for a trigger to action [34,41]. This is supported in studies of individual behavior change across a variety of health behaviors [42-46]. For many patients, hospitalization is often caused by an exacerbation of 1 or more conditions. We assert that hospitalization can serve as the necessary trigger that engages these patients in managing their care [34,41]. In other areas, times of acute crisis have been linked to a greater perception of risk and increased focus on health behaviors [42,43,45,47]. Therefore, hospitalization may create a window of higher motivation to engage, to initiate behavior change, and to foster interest in tools such as IPPs for managing health.

The use of IPPs may come to redefine how patients experience care in the hospital setting, and the use of this new technology may represent a paradigm-shifting change in the way care is delivered. Although the hospital experience is often about moving a patient out of crisis, it also represents an opportunity to influence a patient’s assessment of the benefits and barriers to taking action (seriousness and risk) as well as their confidence about being able to accomplish the necessary behavioral change required to achieve the desired consequence (self-efficacy). Patient portals in this setting may provide a means to increase patient self-efficacy during a particularly receptive time that can be continued after they transition to the outpatient environment.

## Appendix

Multimedia Appendix 1 Stata module 1.

Multimedia Appendix 2 Stata module 2.

Multimedia Appendix 3 Stata module 3.

Multimedia Appendix 4 Stata module 4.

Multimedia Appendix 5 Stata module 5.

Multimedia Appendix 6 Statistics for the frequency and comprehensive measures using the first 30 days within an admission.

## Abbreviations

ADT: admission, discharge, and transfer
IPP: inpatient portal
LOS: length of stay
MCB: MyChart Bedside
MRN: medical record number
OSU: Ohio State University
